# Effect of silver nitrate and growth regulators to enhance anther culture response in wheat (*Triticum aestivum* L.)

**DOI:** 10.1016/j.heliyon.2021.e07075

**Published:** 2021-05-28

**Authors:** Mirza Fida Hassan, S.M.Shahinul Islam

**Affiliations:** Plant Biotechnology and Genetic Engineering Lab., Institute of Biological Sciences, University of Rajshahi, Rajshahi 6205, Bangladesh

**Keywords:** Anther culture, Albinism, Chemical stress pre-treatment factors, Silver nitrate, Plant growth regulators, Regeneration, Wheat

## Abstract

Application of chemical substances as stress pre-treatment factors may positively influence androgenetic responses in cereal and other crops. AgNO_3_ is an anti-ethylene compounds that played a significant role in combination with other chemicals for anther culture responses in cereal and other crop plants. For this study two local wheat cultivars *viz*. Kheri and Akbar were considered to evaluate the effect of AgNO_3_ and to optimize the suitable doses of plant growth regulators, amino acids and sucrose that supplemented in MS medium. Data were recorded on the basis of embryoids induction, regenerated green and albino plants. The results clearly stated that anther culture responses and its major outcomes on regeneration significantly increased with suitable dosages of chemicals. The most noteworthy increases embryo like structures and regenerated green plants accomplished by utilizing the combined effect of AgNO_3_ (50 mg/l) and as plant growth regulators IAA (1.0 mg/l) + kinetin (0.5 mg/l). Best embryo like structures (79.17%) and green plants (33.33%) were recorded in Kheri. The results clearly stated that reducing albinism and increasing embryos induction and green plants 50–75 mg/l silver nitrate along with optimum doses of IAA and kinetin showed very effective results in wheat.

## 1 Introduction

Wheat is one of the most important cereal crops in the world and also in Bangladesh. Its cultivation plays a vital role in livelihood and maintenance of the huge population in developing countries. Plant tissue culture techniques provide a promising and feasible approach to develop various stress tolerant plants. Some stresses such as temperature (physical) and carbon (chemical) starvation enhanced androgenetic responses in crop plants [1, 2]. Efficient plant regeneration in wheat and other cereal crops has been reported to be highly genotype dependent, media components and its dosages, suitable plant growth regulators, culture and growth conditions, etc. [2, 3]. The most common components or factors that significantly influence the gametophytic responses depends on donor plants and its growth conditions either in the field or in greenhouse, physical and chemicals stress pre-treatment factors, sterilization procedures, light, carbon sources, etc. [4, 5, 6, 7]. Anther or microspore culture in wheat has a few impediments for haploid and double haploids production, like low response genotypes, high frequencies of albinism, and extensive time of the actuating and regenerating procedure [[8], [9], [10], [11], [12]]. Till now for androgenetic studies albinism is a great problem specially for cereal crops. To overcome the problem some chemical and physiological stress pre-treatments factors may influence that need to be identified. To overcome the problem microspores needed a better environment so that the physiological limitations could be partially compensated with readily available nutrient resources [4].

Anther culture has been in point of fact developed for production of haploid and doubled haploid lines which are on average a basic procedure for androgenesis in wheat and maize [13, 14, 15]. It has been reported that microspore developmental stage and some chemical substances in addition to the culture medium influenced the efficacy of embryogenesis in cereal crops [16]. Moreover, plant growth regulators have been used in induction media that helped for increasing the ability of embryogenesis in anther culture of wheat, rice and barley [17, 18, 19]. Some chemical substances have frequently been reported to prevent accumulation of phenolic compounds and their toxic effects on plant tissue culture phenomenon. Using silver nitrate, AEC, azetidine, plant growth regulators, gelling agents and culture conditions may positively influenced androgenetic responses in plant species [20, 21, 22, 23, 24].

*In vitro* callus induction, shoot regenerations are interrupted by a vaporous plant hormone like ethylene and its impact goes in the plant cell and tissue culture medium that negatively affect the success of regeneration rate. It has been reported that the silver nitrate established to be a significant instrument in regulating the generation or activity of ethylene to the *in vitro* development [22]. The consideration of auxin or cytokinins in the anther culture medium might be essential for embryo formation in numerous species and other plant growth regulators have rarely been used. There are some proofs that ethylene might be engaged with embryogenesis in plants [25, 26]. AgNO_3_ is an inhibitor of ethylene action in plants which increased the embryos induction marginally [27, 28, 29]. The increase of embryos production through consideration of the ethylene opponent as AgNO_3_ in addition to the culture medium showed significant results in *Brussels* [30]. AgNO_3_ was also used to improvement shoot organogenesis through *in vitro* culture of cassava [31]. In case of maize callus culture AgNO_3_ in addition to the medium showed improved plant regeneration efficiency [32, 33]. Opponents of ethylene biosynthesis have been seemed to stimulate embryogenesis in cultured tissues from a wide level of plant species, for instance, wheat [34], *Solanum* [35] and *Brassica* [36]. Haploid plants production was increased by AgNO_3_ and 2,4-D for durum wheat × maize crosses [37]. The addition of silver nitrate as ethylene inhibitor, in anther culture media has enriched and enhanced embryoids formation in wheat [34, 38]. Microspore embryogenesis was increased by silver nitrate was reported for wheat and *Nicotiana tabacum* [39]. The target of this examination was to test the impact of AgNO_3_ and plant growth regulators on embryoids induction and green plant regeneration and also reducing albino plants. The main attempts were undertaken to optimize the suitable doses of AgNO_3_, amino acids, sucrose and PGRs using two local wheat genotypes and the protocols optimized under this study may be helpful for further advance research on doubled haploid production of wheat and other cereal crops through biotechnological approaches.

## 2 Materials and methods

### 2.1 Plant materials and growth

Mature seeds of two local wheat cultivars *viz*. Kheri and Akbar were taken for its better performance on androgenic responses in a previous study conducted by Mirza Fida Hassan for his PhD work. Plants were grown during winter time (November 2018–February 2019, day time filed temperature was around 20 °C–22 °C) at Robi season in the research field of the Institute of Biological Sciences, University of Rajshahi, Bangladesh.

### 2.2 Harvesting spikes, cold pre-treatment and sterilization

Spikes containing anthers with microspores at the mid-late uninucleate stage were harvested from the donor plants and pre-treated by cold at 4 °C in the dark for 3–15 days. After cold pre-treatment, the spikes inside flag leaves were surface sterilization with 70% (v/v) ethanol for 1 min and rinsed 3–4 times with sterile distilled water. Sterilized anthers were carefully removed from the middle zone of the spikes and inoculated them to semi-solid induction MS medium [40].

### 2.3 Effect of AgNO_3_ and PGRs on embryoids induction and regeneration

The induction medium consisted of MS major salts (modified) in addition with phytohormone as 2,4-D (1.5 mg/l), as amino acids- L-proline (500 mg/l), L-glutamine (500 mg/l), L-aspargine (50 mg/l) and as carbon maltose (90 g/l) was used. Sixteen different concentrations of silver nitrate and plant growth regulators either single or in combinations (treatments) were used for this study. The treatments were, T_1_ = AgNO_3_ (25 mg/l), T_2_ = AgNO_3_ (50 mg/l), T_3_ = AgNO_3_ (75 mg/l), T_4_ = AgNO_3_ (100 mg/l)_,_ T_5_ = AgNO_3_ (25 mg/l) + IAA (0.5 mg/l), T_6_ = AgNO_3_ (50 mg/l) + IAA (1.0 mg/l), T_7_ = AgNO_3_ (75 mg/l) + IAA (1.5 mg/l), T_8_ = AgNO_3_ (100 mg/l) + IAA (2.0 mg/l), T_9_ = AgNO_3_ (25 mg/l) + kinetin (0.25 mg/l), T_10_ = AgNO_3_ (50 mg/l) + kinetin (0.5 mg/l), T_11_ = AgNO_3_ (75 mg/l) + kinetin (0.75 mg/l), T_12_ = AgNO_3_ (100 mg/l) + kinetin (1.0 mg/l), T_13_ = AgNO_3_ (25 mg/l) + IAA (0.5 mg/l) + kinetin (0.25 mg/l), T_14_ = AgNO_3_ (50 mg/l) + IAA (1.0 mg/l) + kinetin (0.5 mg/l), T_15_ = AgNO_3_ (75 mg/l) + IAA (1.5 mg/l) + kinetin (0.75 mg/l), and T_16_ = AgNO_3_ (100 mg/l) + IAA (2.0 mg/l) + kinetin (1.0 mg/l). MS medium without silver nitrate and/or growth regulators considered as the control (Cont. = MS0). The p^H^ of all medium was adjusted to 5.8 and the medium was solidified with 0.85% (w/v) agar (Sigma-Aldrich). For each Petri dish (60 mm) around 45 anthers were inoculated and five petri dishes were considered for each treatment. Then the petri dishes were sealed with parafilm and incubated them at 26 ± 1 °C in dark for embryos induction.

### 2.4 Effect of AgNO_3_ and PGRs on callus induction and regeneration

After 3–4 weeks of culture initiation embryo like structures (ELS) were assessed when its size was 0.5–0.75 mm and transferred them to regeneration medium (MS + NAA- 0.50 mg/l, kinetin- 0.50 mg/l, phytagel- 4 g/l and sucrose- 20 g/l) for shoot and root development. For regeneration each culture vessel (12 cm × 4.5 cm) contained 5–6 embryos and 12 bottles were considered for one replication. Then cultures vessels were incubated for 5–6 weeks at 26 ± 1 °C under 16 h light intensity of 2500 lux.

### 2.5 Data recording parameters

Data were recorded on the basis of embryogenesis and regeneration on the following traits: production of embryo like structures (ELS), expressed as the number of embryos per 100 anthers (ELS/100 anthers); green plant regeneration (GPR); expressed as the number of green plantlets per 100 embryos (GPR/100 ELS); albino regenerated plants (ARP); expressed as the number of albino plantlets per 100 embryos (ARP/100 ELS); and total plants regeneration (TRP); expressed as the number of green and albino plantlets per 100 embryos (TRP/100 ELS).

### 2.6 Rooting and acclimatization of regenerated plants

Well-developed shoots were transferred to ½MS medium supplemented with 1.0 mg/l of NAA, IAA and IBA (phytohormones) + 20 g/l sucrose. For rooting as gelling agent 3.0 g/l phytagel was used. Well-developed rooted plantlets were transferred to pot that contained with peat moss and soil (1:1). Root formation was evaluated on the basis of average number of roots per plants.

### 2.7 Statistical analysis

The cultures were examined weekly and data on regenerated plants was recorded. Analysis of variance (ANOVA) was carried out in order to determine the significance of differences among the treatments. For all cases three replications were undertaken and in a column the mean values followed by same letter(s) are not significantly different at p < 0.05, <0.01 and <0.001, according to Duncan's multiple range tests (DMRT). The DMRT was used to compare the mean performance of the genotypes for ELS formation and regeneration.

## 3 Results

**Figure 1:**
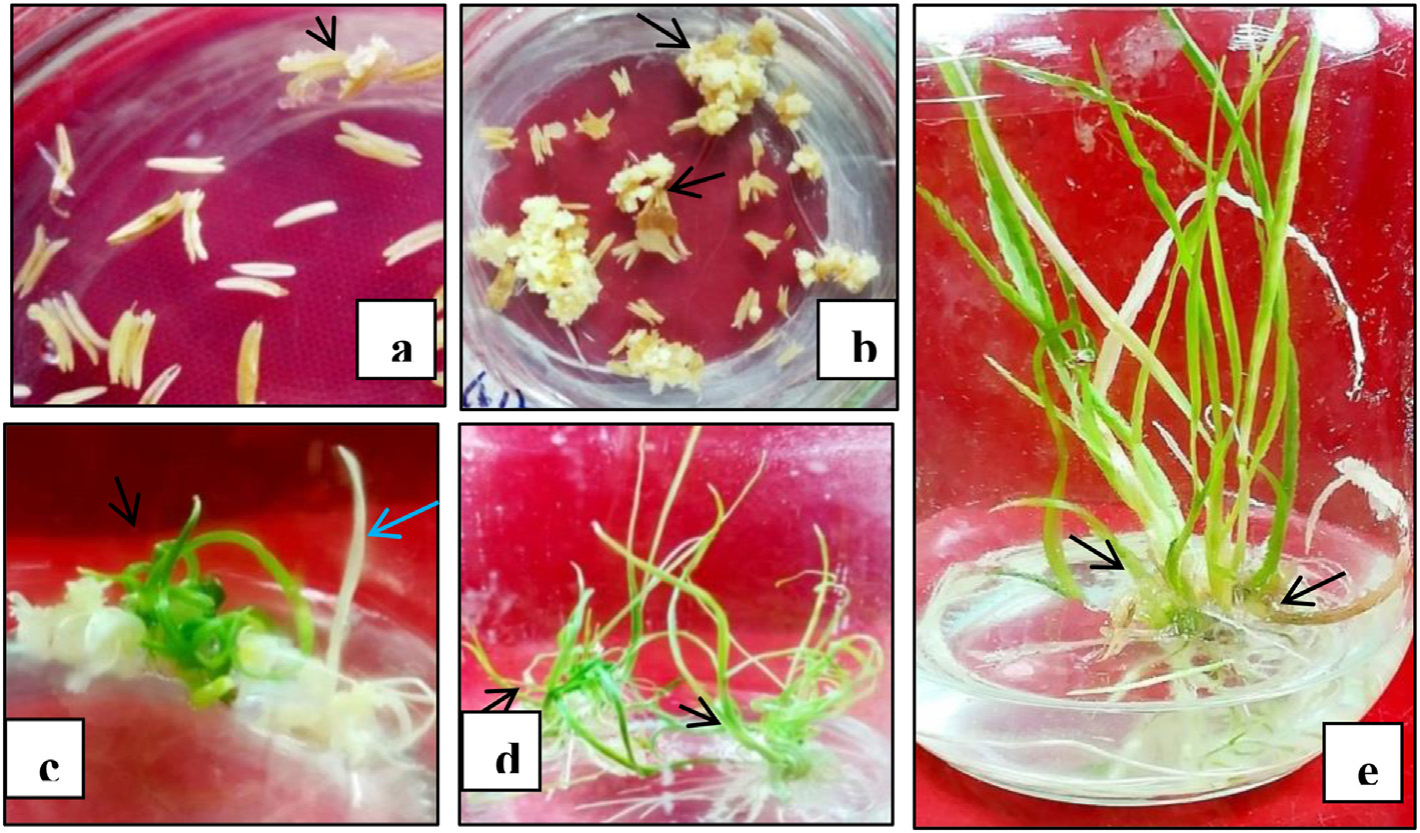
(a–e). Inoculated anthers and its subsequent regeneration. (a) ELS appearance from anthers after two weeks of culture initiation, (b) ELS formation, (c) initiation of green and albino plants, (d) plants with well-developed shoots and roots, (e) well rooted plants were transferred to culture bottle for root and shoot development.

The present investigation was accomplished to find out the effect of different concentrations of silver nitrate and growth regulators either single or in combination on embryoids induction and its subsequent regeneration (Table 1, Figure 1a-e). It was observed that the anthers began to develop in certain size (0.5–0.75 mm) and increasingly the initiation of embryoids induction and regeneration of shoots. The green spots and regeneration was started in calli after 2–3 weeks of culture initiation. Anthers were inoculated on modified MS medium and found some of them turned into brownish which had a high possibility of embryoids formation (Figure 1a). These anthers turned into inflamed and embryo-like structures formed approximately 2–3 w after inoculation and after 2 more weeks, embryos developed at the spherical stage (Figure 1a-b). Some anther derived ELS did not show normal shoot apices and produced hair like structures turned into albino plants (Figure 1c).

**Table 1:** Effect of PGRs and silver nitrate to enhance androgenetic responses in wheat.

Supplements (mg/l)	Treat.	Mean ± SE					
		ELS/100 anthers		GRP/100 ELS		ARP/100 ELS	
		Kheri	Akbar	Kheri	Akbar	Kheri	Akbar
Cont.	-	37.78 ± 2.27fgh	33.89 ± 1.94cd	22.22 ± 2.02cd	19.23 ± 2.22bc	11.25 ± 2.02bcde	8.97 ± 1.28b
AgNO_3_							
25	T_1_	21.67 ± 1.92k	26.67 ± 1.67efgh	10.61 ± 1.52fgh	10.26 ± 1.28fgh	7.58 ± 1.57cde	7.69 ± 2.22b
50	T_2_	32.22 ± 1.94hij	30.28 ± 2.17cdef	11.43 ± 1.65fgh	11.54 ± 2.22efg	5.71 ± 1.65e	6.41 ± 1.28b
75	T_3_	38.61 ± 1.69fgh	28.06 ± 2.47defg	9.17 ± 1.67h	6.41 ± 1.28gh	6.67 ± 1.67de	7.69 ± 2.22b
100	T_4_	32.50 ± 2.20ghij	24.17 ± 1.73fgh	8. 75 ± 1.65h	5.13 ± 1.28h	5.71 ± 1.65e	8.97 ± 1.28b
AgNO_3_ + IAA							
25 + 0.5	T_5_	29.72 ± 1.94j	22.50 ± 1.73gh	21.11 ± 2.22cde	19.23 ± 2.22bc	15.56 ± 2.22ab	12.82 ± 1.28ab
50 + 1.0	T_6_	50.28 ± 2.42cd	41.39 ± 1.47b	28.48 ± 2.19ab	28.33 ± 2.20a	19.39 ± 2.19a	15.83 ± 2.20a
75 + 1.5	T_7_	42.78 ± 2.17ef	25.83 ± 1.27efgh	16.30 ± 1.96def	14.10 ± 1.28cdef	11.11 ± 1.28bcde	8.97 ± 1.28b
100 + 2.0	T_8_	38.89 ± 1.47fg	23.89 ± 2.42gh	17.78 ± 2.57de	11.54 ± 2.22efg	10.37 ± 1.48bcde	10.26 ± 2.56ab
AgNO_3_ + Kinetin							
25 + 0.25	T_9_	28.89 ± 1.69j	23.33 ± 1.73gh	20.00 ± 1.92cde	16.67 ± 2.56bcde	13.33 ± 1.92bc	11.54 ± 2.22ab
50 + 0.5	T_10_	45.83 ± 2.55de	31.39 ± 2.17cde	15.56 ± 1.28efg	12.38 ± 2.52defg	9.63 ± 1.48cde	6.67 ± 2.52b
75 + 0.75	T_11_	36.39 ± 2.00fghi	21.67 ± 1.92h	10.00 ± 1.44gh	6.06 ± 1.52gh	7.50 ± 1.44cde	7.58 ± 1.52b
100 + 1.0	T_12_	30.56 ± 1.69ij	14.72 ± 1.69i	11.11 ± 1.11fgh	8.89 ± 2.22fgh	8.89 ± 2.22cde	8.89 ± 2.22b
AgNO_3_ + IAA + Kinetin							
25 + 0.5+ 0.25	T_13_	49.72 ± 1.94cd	42.50 ± 1.92b	25.45 ± 1.05bc	21.48 ± 1.48b	7.88 ± 1.21 cde	9.63 ± 1.96ab
50 + 1.0 + 0.5	T_14_	79.17 ± 1.92a	66.39 ± 2.17a	33.33 ± 2.55a	31.67 ± 1.92a	11.67 ± 1.67 bcd	8.33 ± 1.67b
75 + 1.5+ 0.75	T_15_	62.78 ± 2.27b	35.83 ± 1.92c	22.22 ± 2.00cd	20.00 ± 1.65bc	9.44 ± 1.47 cde	11.43 ± 1.65ab
100 + 2.0 +1.0	T_16_	52.78 ± 2.42ac	33.89 ± 1.94cd	20.00 ± 2.31cde	18.10 ± 1.90bcd	8.67 ± 1.76 cde	9.52 ± 2.52ab

Control = without AgNO_3_ and PGRs, SE = standard error, ELS = embryo like structures, GRP = green regenerated plants, ARP = albino regenerated plants. In a column the mean values followed by same letter (s) are not significantly different at p ≤ 0.05 according to DMRT.

### 3.1 Effect of PGRs on embryoids induction and regeneration

Excised anthers of two wheat cultivars *viz*. Kheri and Akbar were cultured on MS basal medium and evaluated their effect on embryoids induction. Different growth regulators (kinetin and IAA) either single or in combination with various concentrations of AgNO_3_ was used in addition to the medium. Embryogenesis happened in all concentrations of the growth regulators tested with significant differences in mean number of ELS formation (Table 2). After 3–4 weeks of inoculation, the best ELS were found in Kheri (79.17%) and in Akbar (66.39%) which cultured in MS medium supplemented with IAA (1.0 mg/l) and kinetin (0.5 mg/l). It was observed that in combination with 2,4-D and IAA (1.0 mg/l) showed better performance on embryoids induction (50.58%). Kinetin in addition to the medium showed less effective than 2,4-D and IAA (Table 1). But in combination with IAA and kinetin showed best performance on embryoids induction for all of the studied genotypes. In case of regeneration Kheri (33.33%) and Akbar (31.37%) acquired best performance with IAA (1.0 mg/l) and kinetin (0.5 mg/l). Among the tested genotype and PGRs, IAA showed better performances on regeneration (28.48%) than kinetin (20.0%).

**Table 2:** Analysis of variance (ANOVA) subjected to different concentrations and combinations of silver nitrate and PGRs on ELS and regeneration in two wheat genotypes.

Data sources	Source of variation	DF	Mean sum of square	F-value
ELS formation (Table 1)	Variety (V)	1	2992.600	251.547**∗∗∗**
	Treatment (T)	16	923.276	77.607**∗∗∗**
	V × T	16	82.321	6.920**∗∗∗**
	Error	68	11.897	
Total regenerated plants (Table 1)	Variety (V)	1	263.493	18.008**∗∗∗**
	Treatment (T)	16	527.981	36.084**∗∗∗**
	V × T	16	9.865	0.674 ^NS^
	Error	68	14.632	
Green regenerated plants (Table 1)	Variety (V)	1	158.078	14.462**∗∗∗**
	Treatment (T)	16	324.404	29.679**∗∗∗**
	V × T	16	3.674	0.336^NS^
	Error	68	10.931	
Albino regenerated plants (Table 1)	Variety (V)	1	7.147	0.709^NS^
	Treatment (T)	16	48.690	4.829**∗∗∗**
	V × T	16	6.331	0.628 ^NS^
	Error	68	10.083	

∗∗∗ = significant at p 0.001, NS = non-significant.

### 3.2 Effect of AgNO_3_ on embryoids induction and regeneration

Four different concentrations of AgNO_3_ were added to MS medium and its effect on regeneration was evaluated. Here both of the studied genotypes showed quiet good number of embryos and green plant regeneration in Control (Table 1). Results shows in Table 1, the single uses of AgNO_3_ improved the ability of wheat anthers to produce embryo formation and regeneration. It was observed that 50 mg/l AgNO_3_ showed significantly higher embryoids yield and regenerated green plants for both genotypes. The results indicated a significant difference with various concentrations of AgNO_3_ that employed for embryoids formation and regeneration. In response to single use of AgNO_3_ cv. Kheri showed the highest ELS (38.61%) and green plant regeneration (9.17%) where 75 mg/l AgNO_3_ was used. The Kheri showed 32.22% ELS formation followed by Akbar (30.28%) with 50 mg/l AgNO_3_. The cv. Akbar also showed the highest frequency (11.54%) of green plant production followed by Kheri (11.43%) with the same doses of AgNO_3_ (Table 1).

### 3.3 Combined effect of PGRs and AgNO_3_ on ELS and regeneration

**Figure 2:**
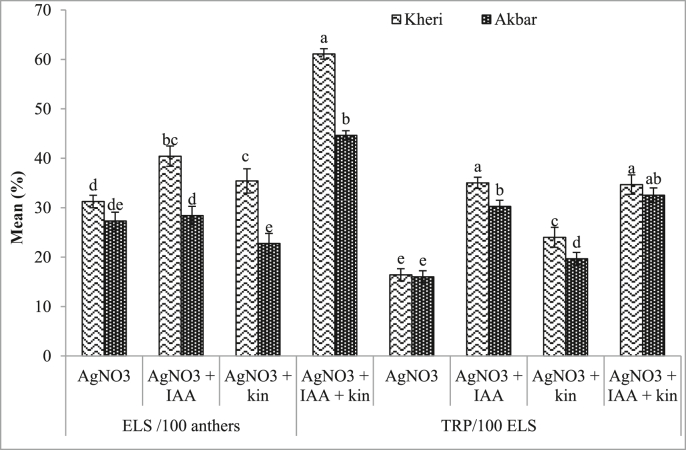
Genotypic effect of ELS formation and TRP on an average of four different concentrations and combinations of silver nitrate and PGRs. In a column the mean values followed by same letter(s) are not significantly different at p ≤ 0.05 according to DMRT.

It was observed that MS in combination with IAA and kinetin exhibits better results than control. In combination with AgNO_3_ (chemicals) and growth regulators (IAA + kinetin) showed better performance on embryoids induction and plant regeneration. It was observed that in addition of AgNO_3_ with various concentrations with IAA + kinetin, the results on ELS and regeneration were increased (Table 1). Table 2 showed significantly effective results on ELS formation and green plant regeneration among the tested genotypes. But interaction with variety and treatments the results showed non-significant on regeneration. On the other hand albino plants were significantly increased for both cultivars with combined effects of PGRs and AgNO_3_ (Figure 1a-e). In combination with silver nitrate and IAA data shows better results than singly used of AgNO_3_. T_6_ showed best performance on ELS for Kheri (50.28%) and Akbar (41.39%) with MS + AgNO_3_ (50 mg/l) + IAA (1 mg/l). For green plant regeneration, T_6_ showed best performance for Kheri (28.48%) and Akbar (28.33%). But AgNO_3_ + kinetin showed lower responses on ELS and regeneration than AgNO_3_ + IAA for both cultivars. Highest embryo yield and regeneration was found in T_13_-T_16_ where AgNO_3_ and as PGRs IAA + kinetin was used. Both cultivars showed best ELS and green plant regeneration with AgNO_3_ (50 mg/l) + IAA (1 mg/l) + kin (0.5 mg/l) in T_14_. The best results on ELS production was recorded for Kheri (79.17%); followed by Akbar (66.39%) with same concentrations and combination of AgNO_3_ and PGRs. Highest green plant regeneration was recorded for Kheri (33.33%) in T_14_ followed by Akbar (31.67%) (Table 1). For each treatment, combined effect of AgNO_3_ and PGRs produced utmost number of ELS and total regenerated green plantlets than single uses. AgNO_3_ (50 mg/l) + kin (0.5 mg/l) + IAA (1 mg/l) showed best ELS and total plant regeneration for Kheri (45.00%) and Akbar (40.00%) (Figure 2). For single uses of AgNO_3_ albino plant shows lower than control (6.42%, 7.69%).

## 4 Discussion

Under this study two plant growth regulators (PGRs) with various concentrations and its efficiency on wheat anther culture responses were evaluated. The results demonstrated that the presence of various PGRs in culture medium showed a vital role on embryoids induction. Single use of four different doses of silver nitrate resulted less number of ELS and GRP than Control (MS0). Silver nitrate in combination with IAA or kinetin singly increased the frequency of ELS and GRP in T_6_, T_7_ and T_10_. Whereas silver nitrate in combination with IAA & kinetin the ELS and GRP were increased 1.5–2.0 fold than Control (T_13_-T_16_). It was observed that albino plants reduced only with AgNO_3_ concentration 50–75 mg/l. In combination with AgNO_3_ and single doses of IAA (T_10_ & T_11_) results showed less number of albino plants in comparison with Control. In another study for wheat and oat anther culture found androgenetic responses could be enhanced in addition with single doses of AgNO_3_ to the culture medium [8, 28]. Hosseini *et al.* [24] reported that the better induction of embryogenesis and direct regeneration of plantlets in maize was obtained by using 2.0 mg/l kinetin and 2.0 mg/l IAA. In the present investigation, this combination of PGRs played a vital role for embryoids induction and plant regeneration. Saidi *et al.* [41] demonstrates a significant role of wheat variety on embryo formation. Yu *et al.* [42] also suggested about BA and NAA combination for promoting embryogenic callus formation. The result obtained by Wang and Wei [17] using somatic cells of wheat is not in agreement with the present study. May be the major causes the explant sources was different. Shah *et al.* [43] observed that the regeneration of wheat plantlets was highest on MS medium supplemented with BAP (2.0 mg/l) in combination with IAA (1.0 mg/l). It is renowned that 2,4-D, commonly applied for callus induction which be able to strongly increase the production of ethylene. Gaseous apparatus of the tissue culture system, especially ethylene, play an important role in growth and development of plants. It has been reported that the embryogenesis and regeneration in callus cultures were inhibited by ethylene [44]. AgNO_3_ is a compelling inhibitor of ethylene activity [27]. Embryo like structure formation and green plantlets development were significantly increased in two wheat genotypes in addition of AgNO_3_ to the media.

In plant tissue culture, the mode of activity of AgNO_3_ is related with the physiological impacts of ethylene, silver particles going about as a focused inhibitor of ethylene activity instead of hindering ethylene amalgamation essentially [37]. The essential addition in embryo formation of the two genotypes in addition with AgNO_3_ up to 50 mg/l stands out from the consequences of Dunwell [29] who worked with *Nicotiana tabacum* anther culture. Lashermes [34] likewise reported a noteworthy improvement in the androgenetic capability of wheat anthers when they were refined on media containing 0.005 g/l AgNO_3_. Buyukalaca *et al.* [21] reported that 10 mg/l AgNO_3_ showed better (2.3–22.9%) embryos formation in *Capsicum*. At the point when the AgNO_3_ concentration was increased from 10 to 15 mg/l the embryo yield was dramatically improved from 1.3-45.7 fold. Increasing AgNO_3_ concentration up to 20 mg/l gave no embryos. Numerous reports have exhibited the constructive outcome of AgNO_3_ on plant tissue culture, in spite of the fact that in certain plants and tissue types no positive or even an inhibitory impact has been reported [36]. The utilization of AgNO_3_ has decreased or restrained rooting of the regenerated shoots [45]. Cristea *et al.* [22] suggests that the slight regeneration frequency were found in the control (without AgNO_3_) and it might be related with ethylene production by the *in vitro* cultured cells or tissues. The inclination of the high concentration of AgNO_3_ to improve the embryogenic response was not clear in some varieties perhaps because of the inhibition of embryo develop at abnormal amounts of silver nitrate. Dias and Martins [20] mentioned comparative objective facts and proposed that some plant either delivered lower dimensions of endogenous ethylene or were less touchy to ethylene for developing life enlistment. The valuable result of silver nitrate on gametic embryogenesis in the present examination is also upheld by comparative investigations on the androgenesis of maize [46], wheat [8], oat [28] and strawberry [23].

In this case combined effect of AgNO_3_ and PGRs (IAA + kinetin), showed higher responses on ELS formation and green plant regeneration than others assortment. Using PGRs in combination demonstrated better results than control, but for ethylene activity, it's not sufficient. Under this study in addition of AgNO_3_ + IAA + kinetin, the result on ELS and regeneration was increased. The maximum frequencies of green plantlets (33.33% and 31.67%) were recorded in presence of 50 mg/l AgNO_3_ in addition to MS medium with 1.0 mg/l IAA and 0.5 mg/l kinetin for Kheri and Akbar respectively. These outcomes were in agreement with Geetha *et al.* [47]. They demonstrated that high frequency of creamy, greenish and nodular callus was obtained in case of leaf as explants sources of *Solanum nigrum* that cultured on MS medium supplemented with NAA (2.0 mg/l) + BAP (0.5 mg/l) + AgNO_3_ (0.2 mg/l). Zhang *et al.* [31] reported that the shoot development was increasing by the use of 1–12 mg/l AgNO_3_ in cassava plant. This investigation considered the improvement of wheat anther culture responses on embryoids induction and green plant regeneration; however use of DH innovation in breeding process needs to get haploid plants. Hence, in future works, it will be important to differentiate many other cultivars and media supporting the androgenetic capability of wheat in Bangladesh.

## 5 Conclusions

Optimization of media, chemical composition and proper dosages of each component is very important factors for success on *in vitro* doubled haploids production that influences androgenetic responses in cereal and other crops. Under this study as chemical stress pre-treatment factors silver nitrate showed a vital role on anther culture response of two Bangladeshi wheat genotypes. Out of four doses 50 mg/l silver nitrate and the optimum concentration of growth regulators (1.0 mg/l IAA and 0.5 mg/l kin.) showed best performances on embryoids yield and green plant regeneration. Interestingly the albinism was reduced when 50–75 mg/l silver nitrate was used either single or in combination with PGRs. The ploidy level of those plants obtained from anther culture system of this study was not analyzed which was not so relevant of this experimental findings. The present study demonstrated that improved anther culture responses due to application of chemical substances with a suitable dose of growth regulators are very important. The idea and methods standardized under this study could be helpful for further advance biotechnological research to wheat and other cereal crops for its improvement.

## Declarations

### Author contribution statement

Mirza Fida Hassan: Performed the experiments; Analyzed and interpreted the data; Wrote the paper.

S M Shahinul Islam: Conceived and designed the experiments; Contributed reagents, materials, analysis tools or data; Wrote the paper.

### Funding statement

This work was supported by the 10.13039/501100001501University Grants Commission of Bangladesh.

### Data availability statement

Data included in article/supplementary material/referenced in article.

### Declaration of interests statement

The authors declare no conflict of interest.

### Additional information

No additional information is available for this paper.
